# Retraining dorsal visual pathways improves cognitive skills and executive control networks following mild traumatic brain injury

**DOI:** 10.3389/fnhum.2025.1698605

**Published:** 2025-12-09

**Authors:** Teri Lawton, John Shelley-Tremblay, Roland R. Lee, Ming-Xiong Huang

**Affiliations:** 1 Cognitive Neuroscience Research and Remediation, Perception Dynamics Institute, Encinitas, CA, United States; 2 Department of Psychology, University of South Alabama, Mobile, AL, United States; 3 Department of Neurology, University of South Alabama, Mobile, AL, United States; 4 Department of Radiology, VA San Diego Healthcare System, San Diego, CA, United States; 5 Department of Radiology, Qualcomm Institute, University of California, San Diego, San Diego, CA, United States

**Keywords:** TBI, cognitive rehabilitation, visual timing, improve cognitive skills, visual working memory, memory recruitment and storage, processing speed, attention

## Abstract

**Background and objectives:**

Mild traumatic brain injury (mTBI) frequently results in persistent cognitive deficits with limited evidence-based rehabilitation options. Visual timing deficits, potentially linked to dorsal visual pathway dysfunction, represent a promising therapeutic target. This study examined whether a novel intervention targeting dorsal visual pathways (*PATH*) demonstrates superior efficacy compared to conventional cognitive therapies targeting ventral pathway deficits or working memory impairments.

**Methods:**

Twenty-four participants (aged 23–62 years) with mTBI were randomized to receive one of three interventions over 12 weeks: (1) *PATH* training targeting dorsal visual pathway function, (2) Orientation Discrimination (OD) targeting ventral pathway pattern discrimination, both of these 20-min interventions followed by 10-min of digit memory exercises, or (3) *ReCollect*, working memory training. Each intervention consisted of 36 sessions (30 min each, three times weekly). Primary outcome was visual working memory (VWM) performance; secondary outcomes included processing speed, reading speed, auditory working memory, selective attention, and cognitive flexibility assessed via standardized neuropsychological measures. Magnetoencephalography (MEG) recordings during resting state and an N-Back task provided neurophysiological validation.

**Results:**

*PATH* training yielded significantly greater VWM improvements (49%) compared to *ReCollect* (13%) and OD (8%) interventions. Repeated-measures ANOVA confirmed superior efficacy of dorsal pathway training (significant at *p* = 0.011). Similar gains for *PATH* training were also found for processing speed, reading speed, and cognitive flexibility, especially when compared to the OD group, revealing importance of strengthening the dorsal stream before digit memory exercises. MEG analysis revealed *PATH*-specific activation in not only bilateral dorsolateral prefrontal cortex, anterior cingulate cortex, posterior parietal cortex, superior occipital gyri, but also the left anterior temporal lobe and hippocampus, primary motor cortex, and the cerebellum, as well as lower gamma-band noise, suggesting enhanced neural timing, attention, working memory, memory consolidation and retrieval mechanisms beyond the targeted dorsal pathways.

**Conclusion:**

Dorsal visual pathway retraining followed by targeted working memory exercises demonstrates superior therapeutic efficacy for cognitive rehabilitation following mTBI compared to conventional approaches. The intervention promotes beneficial neuroplasticity extending to memory-related brain regions, supporting its potential as a targeted, mechanistically-informed therapy for post-concussive cognitive deficits.

## 1 Introduction

Mild traumatic brain injury (mTBI) poses a significant public health challenge, affecting 2.5 million Americans annually (Silverberg et al., 2020). Up to 50% of these individuals experience persistent cognitive deficits that severely impact quality of life and functional independence, yet only one in six concussions is formally diagnosed (Faul et al., 2010). Concussions account for 80% of all traumatic brain injuries, often resulting from motor vehicle accidents, falls, or sports-related trauma (Faul et al., 2010). mTBI presents heterogeneously, and the variability in persistent cognitive deficits across individuals limits the efficacy of existing cognitive training programs (Mavroudis et al., 2024). The majority of persistent post-concussive symptoms are in the cognitive domain (e.g., executive function, attention, working memory) (McInnes et al., 2017). Rehabilitation interventions targeting multiple cognitive domains are needed.

Patients with mTBI frequently suffer from cognitive impairments that hinder return to work or school, and there are currently no proven treatments to address these deficits (Willer and Leddy, 2006; Raymer et al., 2018). Existing options are limited, with cognitive training programs yielding minimal improvements in executive functions and attention (Hallock et al., 2016; Bogdanova et al., 2016). These programs often show inconsistent results and limited transfer to real-world tasks (Buitenweg et al., 2012; Simons et al., 2016). In a large sample of individuals with a history of remote mTBI, Arciniega et al. (2021) found persistent impairments in visual working memory, attention, and executive function, leading to significant daily functioning challenges.

Recent studies suggest that targeted interventions for visual working memory may provide new rehabilitation avenues. Randomized controlled trials indicate that domain-specific computerized cognitive training can significantly improve visual-spatial working memory in patients with acquired brain injuries (Lundqvist et al., 2010). Research has shown that while reference memory can be restored through intensive training after TBI, working memory impairments are more resistant, highlighting the need for novel, mechanism-based approaches (Björkdahl et al., 2013). These findings underscore the urgent need for innovative cognitive rehabilitation strategies that address the vulnerabilities of the visual working memory system in TBI.

### 1.1 Theoretical framework: visual timing deficits and dorsal stream dysfunction

Visual timing deficits, linked to magnocellular impairments, persist in mTBI patients (Poltavski et al., 2017; Lawton and Huang, 2019; Lawton, 2025) and affect dorsal pathways and attention networks. Cognitive control deficits may arise from these underlying neural timing issues, leading to increased distractibility and other symptoms, including fatigue and anxiety (Marshall et al., 2012).

This study posits that remediating foundational visual timing deficits—specifically motion discrimination at low levels of cognitive processing—can improve higher-level cognitive functions. The middle temporal cortex (MT) is critical for motion discrimination (Felleman and Van Essen, 1991) and connects to the prefrontal cortex via vulnerable white matter tracts (Knight, 2007). The dorsal visual stream, composed of predominantly magnocellular neurons (Livingstone and Hubel, 1988; Merigan and Maunsell, 1993) processes spatial information and is integral to both dorsal attention (Vidyasagar, 2013) and working memory (Menon and Uddin, 2010) networks. We propose that training low-level dorsal stream processes through movement discrimination can counteract cognitive declines related to mTBI.

### 1.2 Innovation: perception attention therapy (*PATH*) to reading/insight™ neurotraining

*PATH* neurotraining is a patented intervention (Lawton, 2015) designed to activate the dorsal stream at both low and high processing levels. It enhances visual timing and movement discrimination while also improving attention, processing speed, and working memory (Lawton, 2016, 2025; Lawton et al., 2022). Unlike conventional cognitive training, which shows limited transfer to untrained tasks, *PATH* has demonstrated significant cognitive improvements in both mTBI patients (Lawton and Huang, 2019; Lawton, 2025) and older adults (Lawton and Stephey, 2009; Lawton et al., 2023).

Our previous research indicates that improving low-level visual timing deficits enhances higher-level cognitive functioning, evidenced by both behavioral assessments and magnetoencephalography (MEG) recordings. *PATH* training yielded substantial improvements in visual working memory (*d* = 1.16) (Lawton and Shelley-Tremblay, 2017), surpassing previous meta-analyses (*d* = 0.2 across 115 studies with 2,104 participants) (Rohling et al., 2009). The *PATH* program’s adaptive design, increasing complexity by transitioning from slower to faster movements, optimizes brain training effectiveness (Noack et al., 2009; Simons et al., 2016). Furthermore, evidence indicates that cognitive improvements following *PATH* training are sustained over time (Lawton, 2011; Lawton et al., 2023).

### 1.3 Neurophysiological validation and brain plasticity

MEG recordings provide biomarkers for mTBI-related cognitive improvements (Huang et al., 2014a,b, 2019; Huang C. W. et al., 2016; Huang M. et al., 2016), enabling precise measurement of functional changes in different cortical areas following intervention training with unprecedented accuracy. Our studies confirm that *PATH* training enhances cognitive skills and brain function in mTBI patients (Lawton and Huang, 2019; Lawton, 2025), demonstrating the potential for neurophysiological reorganization (Hillary and Grafman, 2017). Our study is uniquely positioned to determine whether *PATH* neurotraining can address the cognitive deficits frequently observed in mTBI patients, which results from the brain’s improved synchronization as measured by stronger N-back evoked responses or utilization of available detour pathways in a post-training vs. pre-training comparison.

Previous studies showed that gray matter is vulnerable to diffuse axonal injury in mTBI, which damages GABA-ergic inhibitory interneurons (see references in Vascak et al., 2018). GABA-ergic interneurons regulate the activity of neural networks through GABA-ergic inhibition of local excitatory neurons, and generate gamma oscillations (30–80 Hz) through synchronous activity (Cardin et al., 2009; Carlén et al., 2012; Fries, 2009; Sohal et al., 2009; Traub et al., 1996). Animal studies demonstrate that dysfunction or injury to GABA-ergic interneurons causes disinhibition in the neural network by: (1) reducing the synchronization of evoked responses, and (2) increasing spontaneous gamma activity, so-called gamma-band noises, due to absent inhibition of excitatory neurons (Carlén et al., 2012; Cho et al., 2015; Del Pino et al., 2013; Kalemaki et al., 2018; Korotkova et al., 2010). We hypothesize that *PATH* training will improve synchronized brain responses, evoked during MEG working memory N-back task, and reduce gamma-band noise during resting-state MEG exam, reflecting the impact of GABA-ergic interneuron injury in mTBI (Huang et al., 2020).

### 1.4 Preliminary evidence and study rationale

A preliminary investigation (Lawton, 2025) found that visual timing-targeted training (*PATH*) outperformed conventional methods in improving cognitive skills in mTBI patients. Results showed that visual working memory improved by 35% in the *PATH* group, significantly higher than the gains in control groups using conventional methods. The *PATH* group also exhibited marked improvements in auditory working memory, processing speed and attention compared to conventional methods. Neuroimaging revealed enhanced activation in key brain regions, supporting the broader therapeutic impact of *PATH* followed by targeted digit memory (DM) exercises for improving not only vision (superior occipital gyri), attention (anterior cingulate cortex), and executive control (posterior parietal cortex and dorsal lateral prefrontal cortex) networks, but also memory consolidation and retrieval (hippocampus and anterior temporal lobe), as well as primary motor cortex. These findings led to the filing of a provisional patent (Lawton, 2025).

### 1.5 Current study objectives

Building on preliminary findings, this study aims to provide definitive evidence for *PATH*+DM neurotraining as an effective cognitive rehabilitation intervention for mTBI patients. We hypothesize that targeting foundational visual timing deficits will lead to superior cognitive improvements compared to conventional therapies, with corresponding neurophysiological changes measurable through MEG recordings, using expanded imaging protocols. *PATH*+DM training represents a paradigm shift in rehabilitation methodology by directly targeting the underlying etiology of visual timing deficits in mTBI rather than addressing symptoms in isolation. By improving visual timing, this intervention enables utilization of the most efficient working memory networks (Menon and Uddin, 2010), ultimately enhancing post-mTBI quality of life. Our research addresses the critical need for evidence-based, mechanistically-informed cognitive interventions in mTBI rehabilitation, positioning *PATH*+DM training as a transformative therapeutic approach.

## 2 Materials and methods

### 2.1 Study design and ethics approval

This randomized controlled trial was approved by Pearl Institutional Review Board (IRB) and conducted in accordance with the Declaration of Helsinki. All participants provided written informed consent prior to study procedures. The study protocol was registered on ClinicalTrials.gov (NCT03655782). No FDA approval was required, as the Pearl IRB determined these cognitive interventions pose minimal risk to participants and are classified as cognitive therapy rather than medical treatment for TBI. Data safety was monitored throughout the study duration.

### 2.2 Participants

#### 2.2.1 Recruitment and eligibility

Participants with mild traumatic brain injury (mTBI) that occurred at least 3 months earlier (chronic mTBI), aged 18–60 years were recruited through multiple clinical and community sources, including neurologists (Dr. Alan Shahtaji, UCSD Concussion Clinic; Dr. Mohammed Ahmed, Kaizen Brain Center), neuropsychologist Dr. Shaul Saddick, primary care physician Dr. Maysa Nagi, OTR Sarah Kalina, the UCSD clinical trials website, brain injury specialists who taught a California-sponsored Acquired Brain Injury class (Heike Kessler-Heiberg and Brandi Bass), Spine and Sport Physical Therapy, and the San Diego Brain Injury Foundation (SDBIF).

#### 2.2.2 Sample characteristics

**FIGURE 1:**
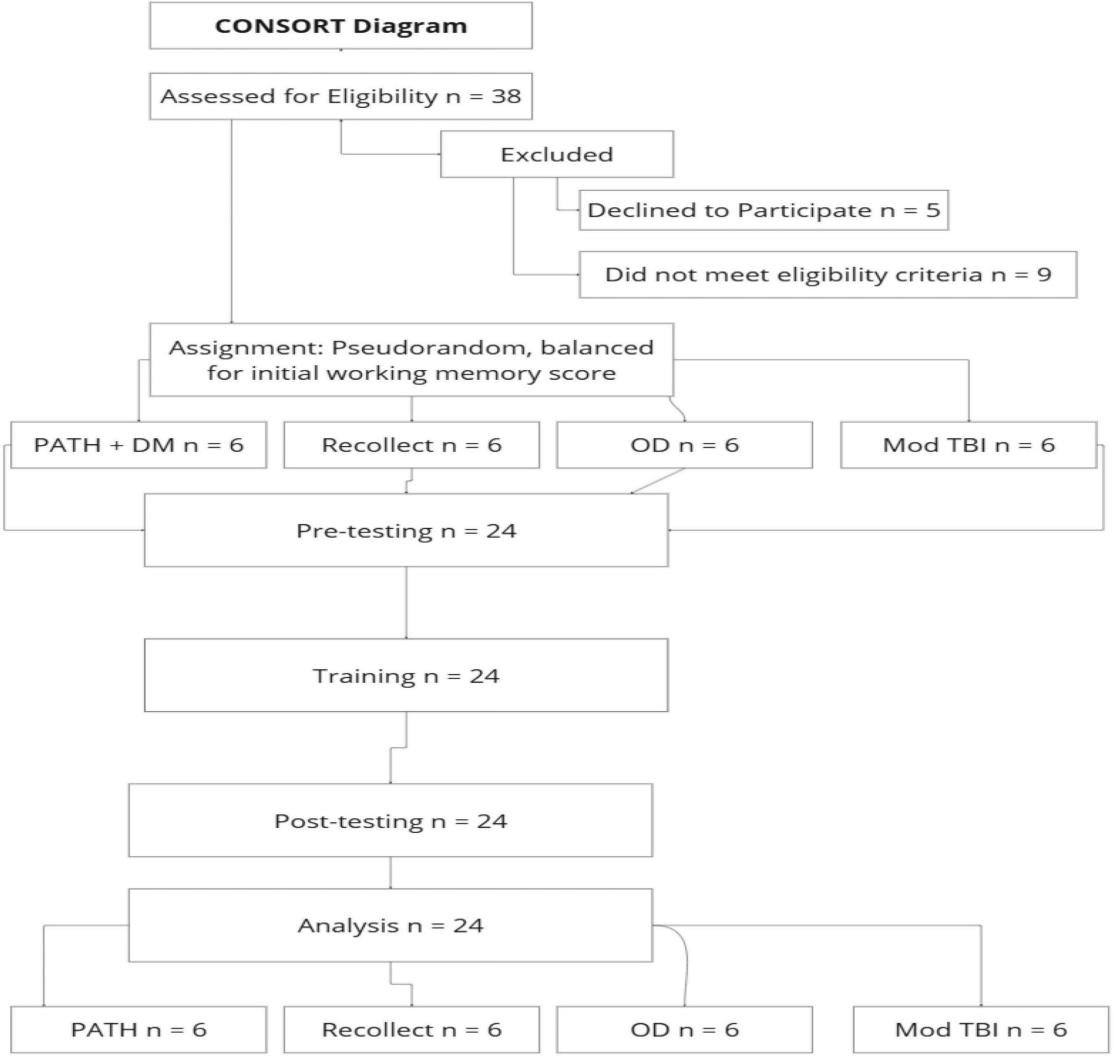
CONSORT diagram for study enrollment, randomization, and analysis.

A total of 38 participants were assessed for eligibility. Following exclusions (*n* = 5 declining participation; *n* = 9 failing eligibility criteria), 24 participants were enrolled and completed the study (see Figure 1). The final sample included 24 participants (9 male, 15 female) aged 23–62 years with diverse ethnic representation: 15 White (Non-Hispanic), 3 White (Hispanic), 1 Asian (Non-Hispanic), 1 African American (Non-Hispanic), and 4 More than One Race (1 Non-Hispanic and 3 Hispanic). This sample size substantially exceeds those of preliminary mTBI studies, which included 4 participants (Lawton and Huang, 2019) and 10 participants (Lawton, 2025). Adults over 62 were excluded to minimize age-related cognitive confounds. There were no exclusions on the basis of gender, race, or ethnicity. The mTBI Human Subject Characteristics (Marshall et al., 2012; American Congress of Rehabilitation Medicine [ACRM], 1993) and the Inclusion and Exclusion Criteria were described previously (Lawton, 2025).

#### 2.2.3 Study sites and procedures

The majority of training and assessment occurred at two coordinated sites: (1) UCSD MEG Center/Qualcomm Institute for neuroimaging, and (2) Perception Dynamics Institute (PDI) for neuropsychological testing and intervention delivery. A third testing site was utilized for logistical purposes to accommodate 3 participants who completed the control condition [Orientation Discrimination (OD)]. Training was conducted either in-person at PDI or remotely via Zoom platform. Only 2 subjects from the OD group, 1 from the *ReCollect* group, and 1 from the *PATH* group completed all their training in person. In contrast, 2 *PATH* subjects and 1 Moderate mTBI subject did over half of their *PATH* training in person, using Zoom only when out of town. The remaining subjects completed all their intervention training via Zoom, as they would not have participated otherwise. A series of ANOVAs were run to check for any effect of the Delivery Method on the Primary and Secondary Outcomes. No significant effects emerged between these groups.

### 2.3 Randomization and group assignment

Participants were pseudo-randomly assigned to one of four intervention groups using balanced allocation based on initial working memory scores:

• *PATH*+DM *group* (*n* = 6): Motion discrimination training plus digit memory exercises

• *ReCollect group* (*n* = 6): Conventional working memory training

• *Orientation Discrimination (OD) group* (*n* = 6): Ventral stream control intervention plus digit memory exercises

• *Moderate TBI group* (*n* = 6): Participants with previous moderate TBI, now mild, receiving *PATH*+DM

A fourth group consisting of participants with previous moderate TBI (now classified as mild) was added to explore intervention effects in this population, as this represented the predominant sample referred by SDBIF; all received *PATH*+DM training. Participants in the three treatment groups (*PATH*+DM, Moderate TBI *PATH*+DM, and *ReCollect*) underwent both standardized neuropsychological testing and MEG/MRI recordings to assess neurophysiological changes in dorsal, attention, and executive control brain networks following intervention. Groups were balanced for age, sex, and baseline scores on visual working memory, auditory working memory, processing speed, and selective attention (verified by one-factor ANOVA, *p* > 0.05).

### 2.4 Outcome measures

#### 2.4.1 Primary outcome

*Visual Working Memory (VWM):* Assessed using the Test of Information Processing Skills (TIPS) standardized percentiles.

#### 2.4.2 Secondary cognitive outcomes

- *Auditory working memory*: Wechsler Adult Intelligence Scale-IV (WAIS-4) Working Memory Index (Digit Span and Letter-Number Sequencing subtasks)
- *Processing Speed:* WAIS-4 Processing Speed Index (Coding and Symbol Search subtasks)
- *Selective Attention:* Delis-Kaplan Executive Function System (DKEFS) Color-Word Interference test (Inhibition subtask)
- *Cognitive Flexibility:* DKEFS Color-Word Interference test (Inhibition Switching subtask)
- *Reading Speed:* Computer-based assessment measuring reading speed using sequential 6-word text segments from “Cheaper by the Dozen,” with presentation speed progressively increased until accuracy drops to 79%.
- *Reading Proficiency:* Assessed using the Adult Dyslexia Test (ADT),^1^ which evaluates visual and phonological processing abilities across a 7-point scale ranging from markedly below normal to above normal.

#### 2.4.3 Quality of life measures

*SF-36 Health Survey:* Validated 36-item measure assessing eight domains of health-related quality of life with established psychometric properties (internal consistency α > 0.80; test-retest reliability 0.60–0.95).

*Rivermead Post-Concussion Symptoms Questionnaire (RPQ):* 16-item measure assessing physical, cognitive, and emotional symptoms rated on 5-point Likert scales (Cronbach’s α > 0.80).

*Brain Injury Visual Symptom Survey (BIVSS):* 28-item measure assessing eyesight clarity, visual comfort, doubling, light sensitivity, dry eyes, depth perception, peripheral vision, and reading symptoms rated on 5-point Likert scales (Cronbach’s α > 0.80).

#### 2.4.4 Neurophysiological biomarkers

*Magnetoencephalography (MEG):* Pre-post recordings during N-back working memory tasks to assess improvements in not only bilateral dorsolateral PreFrontal Cortex (dlPFC), anterior and posterior cingulate cortex, superior occipital gyri, but also the left anterior temporal lobe, primary motor cortex, hippocampus, and cerebellum, and during resting state to assess gamma-band noise.

### 2.5 Interventions

#### 2.5.1 *PATH*+DM training (experimental condition)

**FIGURE 2:**
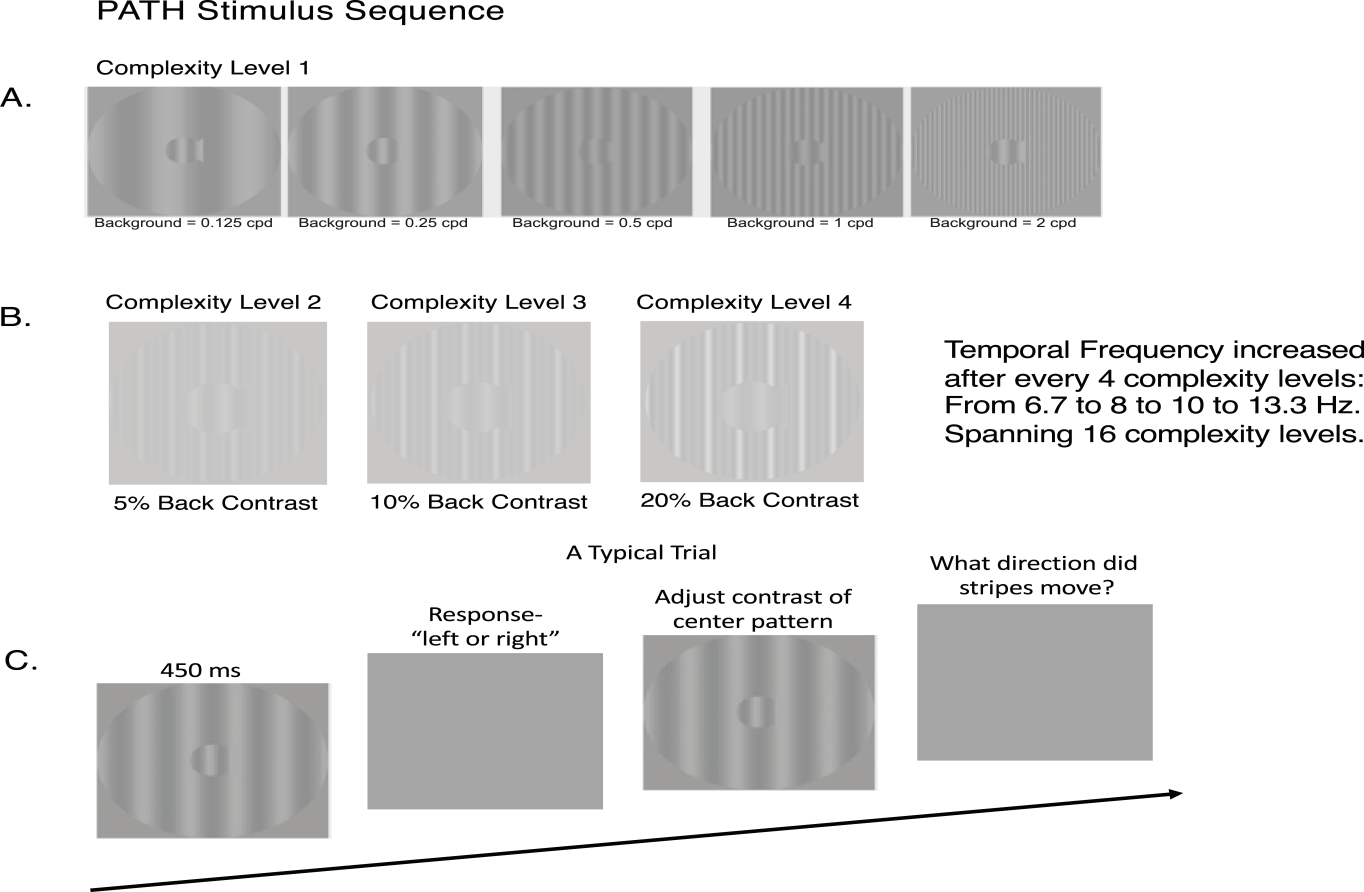
*PATH* stimulus sequence. **(A)** Sample patterns for *PATH* training at Complexity Level 1, showing backgrounds spanning ± two octaves in spatial frequency from the “fish-shaped” test pattern (0.5 cyc/deg). This background set was presented in the same order for each of the four test spatial frequencies (0.25, 0.5, 1, and 2 cyc/deg). **(B)** Complexity levels 2, 3, and 4 display multifrequency backgrounds for the center pattern shown in 2A, maintaining the same fundamental frequency as complexity level 1, with difference frequency equal to the test frequency and increasing background contrast from 5 to 10 to 20%. **(C)** Typical trial sequence for *PATH to Reading*/Insight intervention. Patterns flash on screen for ≤ 450 msec while center stripes move left or right. Following pattern offset, participants respond using left or right arrow keys, with incorrect responses signaled by a brief tone. This sequence continues until contrast threshold is measured using optimized psychophysical procedures (Higgins et al., 1984). Upon completing all 20 *PATH* neurotraining patterns, the program displays completion feedback including: a “Thank You” message, progress stars for each complexity level completed, and threshold function graphs (optimal, current, initial) for each test frequency with corresponding background patterns.

Visual Timing Component (20 min): Participants completed motion direction-discrimination training using the *PATH* web application.^2^ The intervention utilized low-contrast achromatic sine wave gratings moving left or right, specifically designed to activate magnocellular neurons in the dorsal visual stream, relative to stationary background (see Figure 2). A more detailed description of the *PATH* neurotraining intervention was presented previously (Lawton, 2025).

*Training Protocol:*

- Advancing complexity across 16 levels in Motion program, followed by 16 levels in MotionMemory program
- Adaptive contrast thresholds measured using double-staircase procedures (20–40 trials per threshold)
- Real-time motivational feedback including visual progress indicators and performance graphs

The cross-platform *PATH* training web-app: (1) incorporates approaches proven to boost perceptual learning, using patterns designed to stimulate magnocellular neurons (low-contrast achromatic sinewave gratings moving left or right) relative to a stationary background designed to entrain motion discrimination (Lawton, 1984, 1985, 1989), (2) uses motivating tasks (Lawton et al., 2022), and (3) coordinates attention and reinforcement (Lawton, 2011, 2015, 2016; Lawton and Shelley-Tremblay, 2017) into a compelling video game, providing a framework where participants are confident in their performance (Lawton, 2011, 2015, 2016; Lawton and Shelley-Tremblay, 2017) and receive consistent reinforcement to the training stimuli (Lawton et al., 2022).

*Digit Memory Component (10 min):* Following visual training, participants completed working memory exercises presenting digit sequences (5–10 digits) for 500 ms each, each separated by a 500 ms interstimulus interval (ISI), with participants recalling sequences in correct order. The digit quantity increased incrementally from 5 to 10 digits (no repeating digits), approaching the retention limit for a 500-ms interval since serial search requires 20–45 ms per item (Wolfe and Horowitz, 2004) while maintaining previously presented items in working memory.

After sequence presentation, participants wrote down the observed digits in correct order on a blank screen. This targeted practice approach enhances the specific cognitive skill being trained, with previous *PATH* studies demonstrating 11-fold vs. 3-fold improvements when combining visual training with relevant cognitive exercises (Lawton, 2011).

Sequential processing utilizes claustral connections where cross-frequency coupling between low-frequency claustral signals and higher-frequency cortical oscillations enables synchronized neural modulation across brain regions (Vidyasagar and Levichkina, 2019). This coupling mechanism facilitates communication between cortical areas and mediates working memory and learning processes (Canolty and Knight, 2010; Hyafil et al., 2015). The digit memory task is therefore predicted to enhance coupled theta/gamma and alpha/gamma oscillations following *PATH* training, extending the intervention to 30 min total. Consequently, *PATH* training may enhance the evoked responses in regions that are part of typical working memory neurocircuitry during an N-back working memory task.

#### 2.5.2 Orientation Discrimination control (control condition)

**FIGURE 3:**
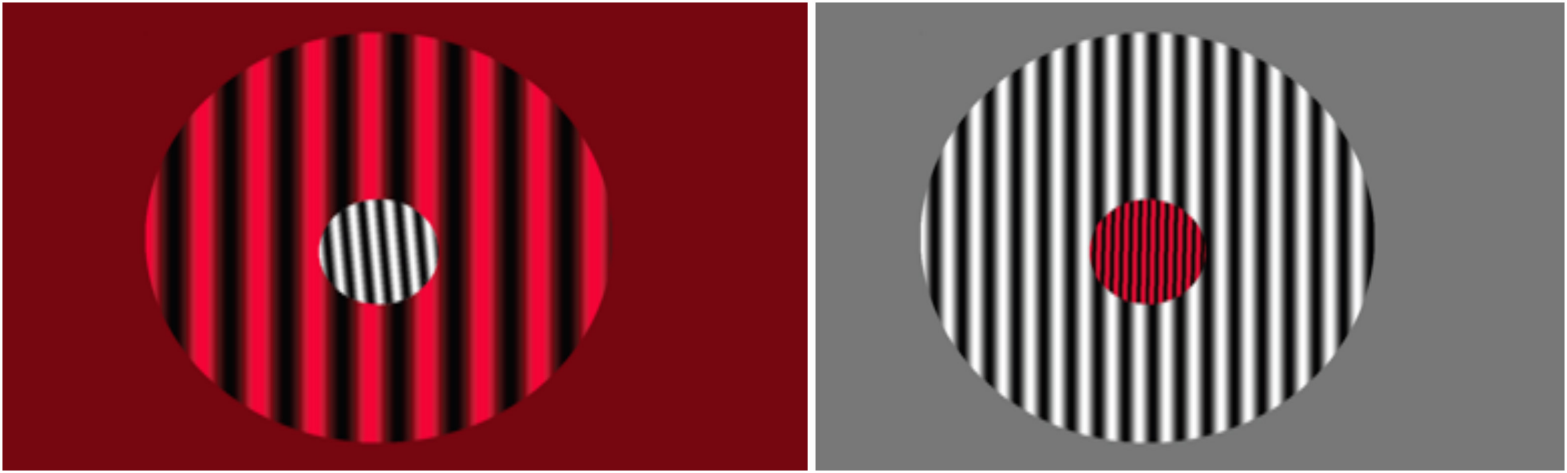
Sample patterns for Orientation Discrimination task.

Identical to *PATH* training except using high-contrast (97%) stationary gratings in multiple colors (red/black and white/black) (see Figure 3), targeting ventral stream pattern discrimination (Kaplan and Shapley, 1986; Ungerleider and Mishkin, 1982) rather than dorsal stream motion processing. Participants discriminated left/right orientation tilts of center pattern relative to vertically striped background pattern, using the same adaptive threshold procedures used in *PATH*, for 20-min, followed by the same 10-min digit memory task. This task was described in detail previously (Lawton, 2025).

#### 2.5.3 *ReCollect* working memory training (comparison experimental condition)

Participants completed 15 min of adaptive N-back training using *ReCollect* software (Pergher et al., 2019; Pahor et al., 2020), requiring comparison of current stimuli to items presented n-steps back in sequence. The task difficulty (level of n) was adaptively adjusted based on individual performance. This was followed by 15 min of Span training involving sequential recall of increasing numbers of colored flowers. The N-back paradigm specifically targets dorsal lateral PreFrontal Cortex (dlPFC) (Pahor et al., 2020), and represents one of the most frequently used working memory training protocols (Pergher et al., 2019).

### 2.6 Training schedule and fidelity

All interventions were delivered 3 times weekly (Monday, Wednesday, Friday) for 12 weeks, totaling 36 sessions of 30 min each. Training data with timestamps were automatically collected and stored securely in HIPAA-compliant cloud databases. Research staff monitored individual progress data to ensure proper task performance and provide strategy coaching when needed.

### 2.7 Neuroimaging procedures

#### 2.7.1 MEG data acquisition

Seventeen participants from treatment groups (12 *PATH*+DM, 5 *ReCollect*) underwent pre-post MEG recordings during: (1) N-back working memory tasks (1-back and 2-back conditions), and (2) Two blocks of 5-min resting-state MEG (rs-MEG) exam with eyes closed (see MEG Task Protocol below). Structural MRI was performed prior to initial MEG recording for anatomical co-registration.

Specifically, MEG responses to the N-back WM task and rs-MEG were collected using the Triux-neo whole-head MEG system (MEGIN/Neuromag, Helsinki, Finland) with 306 MEG channels. Participants were seated in upright position inside a multi-layer magnetically-shielded room (IMEDCO-AG) (Cohen et al., 2002) at the UCSD MEG Center. Data were sampled at 1,000 Hz and were run through a high-pass filter with a 0.1 Hz cut-off, and a low-pass filter with a 330 Hz cut-off. Eye blinks and eye movements were monitored using two pairs of bipolar electrodes with one pair placed above and below the left eye, and the other pair placed on the two temples. Heart signals were monitored with another pair of bipolar electrodes. Precautions were taken to ensure head stability; foam wedges were inserted between the subject’s head and the inside of the unit, and a Velcro strap was placed under the subject’s chin and anchored in superior and posterior axes. Head movement across different sessions was about 2–3 mm on average.

#### 2.7.2 MEG task protocol

During the MEG working-memory task, participants monitored letter sequences (500 ms presentation, 3,000 ms interstimulus interval) and responded to target matches using MEG-compatible response devices. Approximately 50 trials per load condition were collected. The MEG working memory letter-based N-back task and the process of processing MEG responses were described in detail in our previous publication (Huang et al., 2019).

During the resting-state MEG exam, the recordings were divided into two 5-min blocks where the participant was instructed to keep his/her eyes closed and empty his/her mind. The rs-MEG recording was divided into 2.5 s sections. The data in each section were first DC-corrected and then run through a band-pass filter for gamma band (30–80 Hz). Notch filter at 60 Hz was applied to remove the power line signals. More details about the rs-MEG exam were described in our previous study (Huang et al., 2020).

#### 2.7.3 MEG data pre-processing and artifacts removal

MEG sensor waveforms in raw (un-averaged) format were first run through MaxFilter, also known as signal space separation (Taulu et al., 2004a,b; Song et al., 2008), to remove external interferences (e.g., magnetic artifacts due to metal objects, strong cardiac signals, environment noises, etc.). Next, residual artifacts near the sensor array due to eye movements and residual cardiac signals were removed via Independent Component Analysis using Fast-ICA (Hyvarinen, 1999; Hyvarinen and Oja, 2000). The waveforms associated with top independent components (ICs) were examined by an experienced MEG data analyst, along with ECG and EOG signals. ICs associated with eye blinks, eye movements, heartbeats, and other artifacts were removed.

#### 2.7.4 Structural MRI, MEG-MRI registration, boundary element method forward calculation

Structural MRI of the participant’s head was collected using a General Electric 3T MRI scanner. The acquisition contains a standard high-resolution anatomical volume with a resolution of 0.94 × 0.94 × 1.2 mm^3^ using a T1-weighted three-dimensional inversion recovery prepared fast spoiled gradient recalled (3D-IR-FSPGR) pulse sequence. To co-register the MEG with MRI coordinate systems, three anatomical landmarks (i.e., left and right pre-auricular points and nasion) were measured for each participant using the Probe Position Identification system (Polhemus, United States). By using MRILAB (MEGIN/Neuromag) to identify the same three points on the participant’s magnetic resonance (MR) images, a transformation matrix involving both rotation and translation between the MEG and MR coordinate systems was generated. To increase the reliability of the MEG-MR co-registration, approximately 120 points on the scalp were digitized with the Polhemus system, in addition to the three landmarks, and those points were co-registered onto the scalp surface of the MR images.

The T1-weighted images were also used to extract the brain volume and innermost skull surface (SEGLAB software by MEGIN/Neuromag). The Realistic Boundary Element Method (BEM) head model was used for MEG forward calculation (Huang et al., 2007; Mosher et al., 1999). The BEM mesh was constructed by tessellating the inner skull surface from the T1-weighted MRI into ∼6,000 triangular elements with ∼5 mm size. A cubic source grid with a 5-mm size was used for calculating the MEG gain (i.e., lead-field) matrix, which leads to a grid with ∼10,000 nodes covering the whole brain. MRIs were reviewed by a board-certified neuroradiologist (RRL), who determined that no mTBI or healthy control participant had visible lesions on MRI.

#### 2.7.5 MEG data analysis

MEG source imaging was performed using Fast-VESTAL procedures (Huang et al., 2014a, 2019; Huang C. W. et al., 2016) with 2–3 mm spatial resolution. To examine functional changes in synchronized MEG working memory N-back signals, working memory network responses (200–1,000 ms interval) were studied to evaluate the effectiveness of *PATH*+DM vs. conventional *ReCollect* training following the voxel-wise MEG source imaging steps for working memory N-back described in our previous study (Huang et al., 2019).

Similarly, the Fast-VESTAL based source imaging of the resting-state MEG gamma-band activity was performed for mTBI participants under *PATH*+DM or *ReCollect* training following the steps described in our previous study (Huang et al., 2020).

In all participants, voxel-wise whole brain MEG source magnitude images obtained from Fast-VESTAL were first spatially co-registered to the MNI-152 (Grabner et al., 2006) brain-atlas template using a linear affine transformation program, FLIRT, in the FSL (v.7.4.0) software package (Smith et al., 2004; Woolrich et al., 2009). Then in MNI-152 space, the MEG source magnitude images were spatially smoothed using a Gaussian kernel with 5 mm full width half maximum (FWHM), followed by a logarithmic transformation using FSL. Next, voxel-wise group statistical analysis was performed to detect group differences in brain activation during the MEG N-back task as well as the rs-MEG.

Repeated-measures ANOVA assessed group differences (*PATH*+DM vs. *ReCollect*) for treatment effects (post- vs. pre-intervention). In this approach, “ranova” function in MATLAB (MathWorks, Inc., version R2024b) was used for the voxel-wise MEG source magnitude images. Family-wise error across voxels was corrected using standard cluster analysis for the ANOVA maps to control for family-wise errors at a corrected *p* < 0.01 level, using “3dFWHMx” and “3dClustSim” functions in the AFNI,^3^ version 24.3.10. A mask that contained the statistically significant clusters was created, and then applied to the ANOVA maps to create the corrected group statistical maps for the MEG source magnitude images.

### 2.8 Behavioral statistical analysis

All statistical analyses were conducted in R (Version 4.5) using RStudio (Version 2025.09.1+401, “Cucumberleaf Sunflower” Release for Windows) as the integrated development environment. The overall analytic strategy followed a consistent workflow across outcome measures. First, 2 (Time: Pre, Post) × 3 (Group: *PATH*, *ReCollect*, OD) mixed-model ANOVAs were used to evaluate group differences in changes over time. When Time 1 (pretest) scores were equivalent across groups, this mixed-effects ANOVA framework was retained. However, when baseline (Time 1) group differences were present, we conducted a one-way ANCOVA on post-test scores, using the corresponding Time 1 value as a covariate to statistically control for initial non-equivalence. The decision to use standard ANOVA or Welch-type adjustments was based on tests of homogeneity of variance (Levene’s test); when variances were unequal, the more robust ANOVA variant (Welch’s test) was applied. To further examine patterns of change and aid interpretation, one-way ANOVAs on change scores (Post—Pre) and corresponding visualizations with standard error bars were generated. Planned pairwise contrasts between *PATH* and each comparator group were conducted using emmeans, and Cohen’s d effect sizes were calculated to quantify the magnitude of these differences. All mixed-model and ANCOVA analyses were performed using afex, which provides Type III sums of squares and Greenhouse–Geisser corrections as needed. Descriptive statistics and data preparation were conducted using dplyr within the tidyverse, figures were generated with ggplot2 (and ggpubr for significance annotations), and tables were formatted in APA style using flextable and officer. This workflow ensured that each outcome was analyzed with the most statistically appropriate model given its baseline characteristics and variance structure, while maintaining consistency and reproducibility across measures.

### 2.9 Data management and confidentiality

Participant confidentiality was protected through coded identifiers, with the key linking participant names to ID numbers stored in password-protected files on secure computers and locked file cabinets. To minimize bias, all intervention data were automatically collected by software programs, and all behavioral data entry into REDCap database and FITBIR repository, as well as statistical analyses, were conducted by the study statistician who was independent of the intervention delivery. This ensured that the principal investigator remained blinded to results during data collection and analysis.

## 3 Results

### 3.1 Neuroimaging results

**FIGURE 4:**
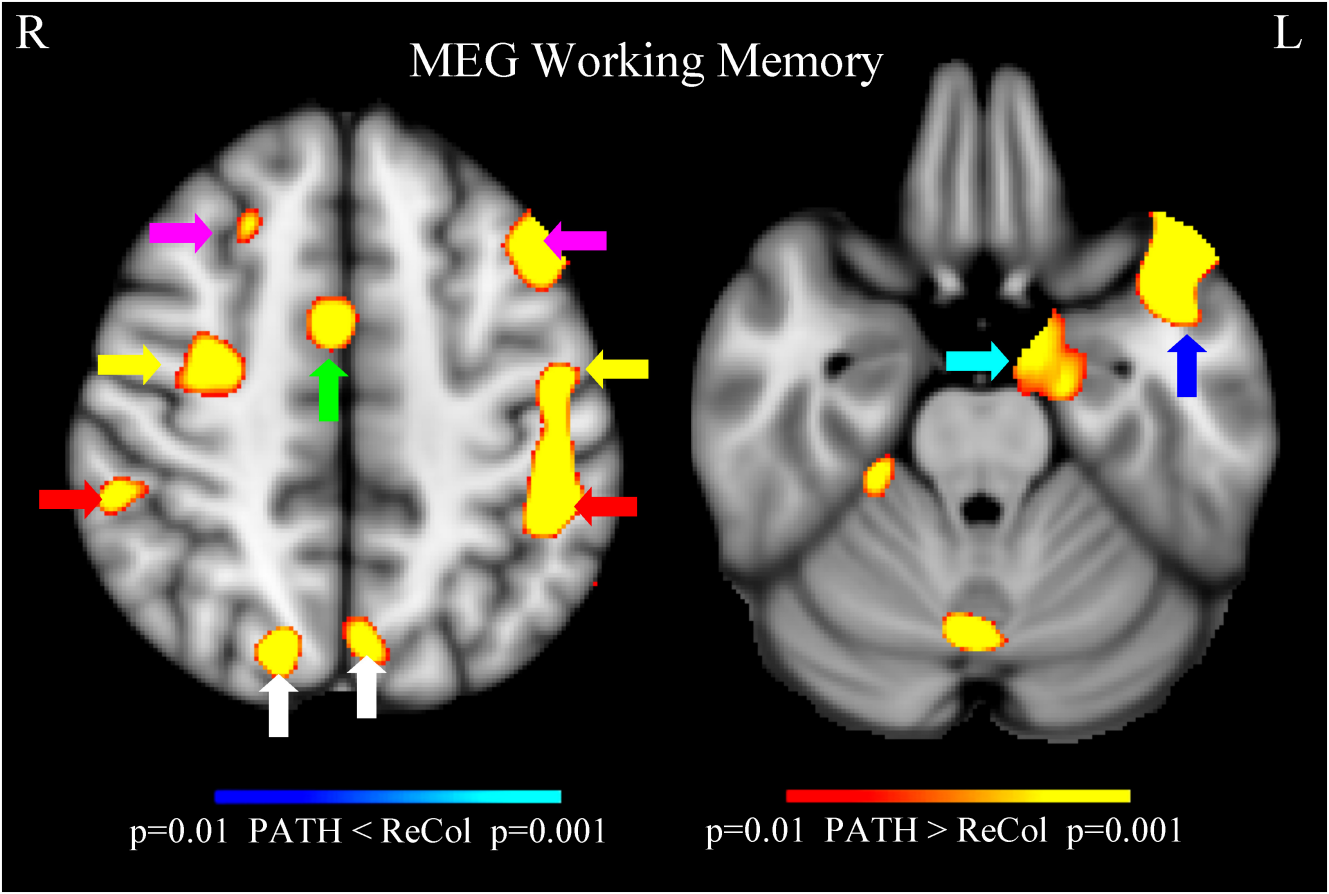
Evoked MEG working memory N-back task showed stronger (post- vs. pre-treatment) responses in individuals with mTBI who finished the *PATH* treatment compared to those who went through the conventional *ReCollect* treatment, significant at *p* = 0.001. Stronger responses were from: bilateral dlPFC (magenta arrows), ACC (green arrows), primary motor cortex (yellow arrows), PPC (red arrows), superior occipital gyri (white arrows), left anterior temporal lobe (blue arrow), and left hippocampus (cyan arrow), and cerebellum.

Evoked MEG working memory N-back task showed stronger (Post- vs. Pre-treatment) responses in individuals with mTBI who finished the *PATH* treatment compared to those who went through the conventional *ReCollect* treatment, significant at *p* = 0.001. Stronger responses were from: bilateral dorsal lateral pre-frontal cortex (dlPFC) [magenta arrows], anterior cingulate cortex (ACC) [green arrows], primary motor cortex (yellow arrows), posterior parietal cortex (PPC) [red arrows], superior occipital gyri [white arrows], left anterior temporal lobe [blue arrow], and left hippocampus [cyan arrow], and cerebellum, as shown in Figure 4. Four cortical areas not previously identified following PATH neurotraining—the hippocampus, anterior temporal lobe, primary motor cortex, and cerebellum—showed significantly improved cognitive function. The other cortical areas in vision (superior occipital gyri), attention (ACC) and executive control (dlPFC and PPC) networks demonstrated stronger Post- vs. Pre-treatment MEG responses, as previously observed after *PATH* neurotraining (Lawton and Huang, 2019; Lawton et al., 2023). The MEG data also showed mTBI patients had stronger working memory evoked responses after *PATH* treatment compared to the *ReCollect* treatment from the typical working memory neurocircuitry (e.g., dlPFC, ACC, PPC, etc.). These findings highlight the potential of *PATH* training for improving cognitive functions. A larger number of subjects will be needed to assess the relative activity of Pre- vs. Post theta, alpha, and gamma activity, since our current sample does not offer adequate power to answer this question.

**FIGURE 5:**
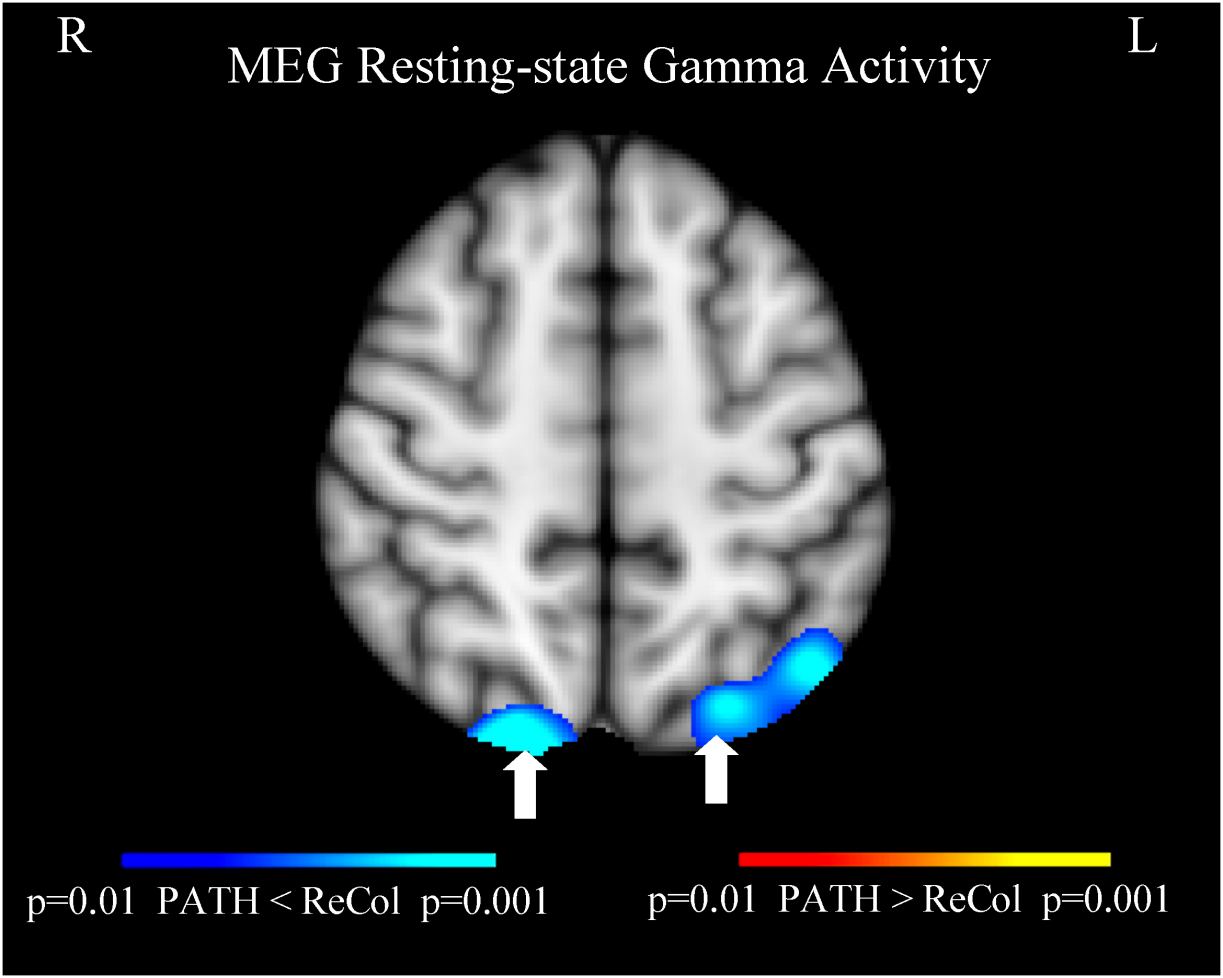
Resting-state MEG activity showed less gamma-band noise in individuals with mTBI who finished the *PATH* treatment than those who went through the conventional *ReCollect* treatment from the bilateral superior occipital gyri (white arrows).

*Result of resting-state MEG gamma-band activity*: Resting-state MEG activity (Figure 5) showed less gamma-band noise in individuals with mTBI who finished the *PATH* treatment compared to those who went through the conventional *ReCollect* treatment. Less gamma-band noise, determined by MEG recordings measuring less noise in gamma-band output, as described previously (Huang et al., 2020), originates from the bilateral superior occipital gyri (indicated by white arrows).

*Baseline pre-treatment MEG responses*: In contrast, the baseline (pre-treatment) working-memory N-back MEG exam did not show significant differences in cortex, analyzed using voxel-wise ANOVA. One region in the right cerebellum showed lower N-back response in mTBI subjects from the *PATH* group than those from the *ReCollect* group (see Supplementary Figure 1). No cortical or sub-cortical regions showed significant difference in resting-state gamma-band noise between these two mTBI groups.

### 3.2 Behavioral results

**FIGURE 6:**
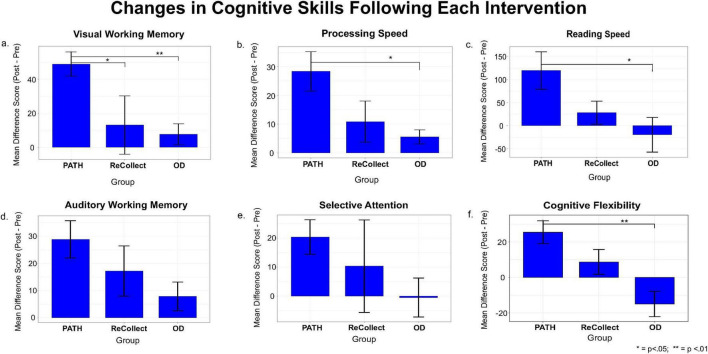
**(a)** Mean difference in visual working memory standardized percentiles (Post—Pre) by group; **(b)** mean difference in Processing Speed standardized percentiles (Post—Pre) by group; **(c)** mean difference in Reading Speed words per minute (Post—Pre) by group; **(d)** mean difference in Auditory Working Memory standardized percentiles (Post—Pre) by group; **(e)** mean difference in Selective Attention standardized percentiles (Post—Pre) by group; **(f)** mean difference in Cognitive Flexibility standardized percentiles (Post—Pre) by group. Error bars indicate ± 1 SEM. *p < 0.05; **p < 0.01; error bars indicate ± 1 SEM.

A correlational analysis was first conducted between Age and all dependent variables. Age was significantly correlated only with the DKEFS Color-Word Interference Test Inhibition Score (DKEFS-Selective Attention), *r*(23) = 0.482, *p* = 0.01. Therefore, DKEFS-Selective Attention was analyzed using an ANCOVA controlling for Age. All other dependent variables were analyzed using 2 (Time: Pre, Post) × 3 (Group: *PATH*, *ReCollect*, OD) mixed-model ANOVAs with Greenhouse–Geisser correction applied as needed. Planned pairwise comparisons were then conducted to test the a priori hypotheses that *PATH* would outperform *ReCollect* and that *PATH* would outperform OD. In order to further analyze and visualize the unique effect of training for each group, a one-way ANOVA was then run on the Pre-Post difference scores and resulting difference plot with standard errors was produced (Figure 6).

Because two subgroups (*PATH* and Moderate TBI) both received the *PATH*+DM intervention, it was necessary to determine whether these groups differed and whether they could be combined. To test this, a series of 2 (Time: Pre, Post) × 2 (Group: *PATH*, Moderate) mixed-model ANOVAs with Greenhouse–Geisser correction were conducted on all primary and secondary outcome measures. Across all measures, the main effect of Group was nonsignificant, indicating no baseline or overall performance differences between *PATH* and Moderate: Visual Working Memory [*F*(1, 10) = 3.50, *p* = 0.091], Auditory Working Memory [*F*(1, 10) = 0.07, *p* = 0.799], Processing Speed [*F*(1, 10) = 2.49, *p* = 0.146), Cognitive Flexibility [*F*(1, 10) = 0.28, *p* = 0.608], Selective Attention [*F*(1, 10) = 1.24, *p* = 0.291], and Reading Speed [*F*(1, 10) = 0.32, *p* = 0.584). Moreover, no Group × Time interactions were significant (all *p* > 0.47), indicating that the pattern of pre-to-post change did not differ between *PATH* and Moderate TBI participants. Given the consistent lack of group differences, the *PATH* and Moderate TBI participants were empirically justified to be combined into a single *PATH*+DM group for all subsequent analyses.

#### 3.2.1 Demographic characteristics

Table 1 presents the final demographic characteristics for each group. The *PATH* group (*n* = 12) had a mean age of 43.2 years (SD = 13.6) and a mean education of 16.2 years (SD = 2.3). The ReCollect group (*n* = 6) had a mean age of 39.5 years (SD = 8.9) and a mean education of 15.7 years (SD = 2.3). The OD group (*n* = 6) had a mean age of 38.7 years (SD = 16.3) and a mean education of 14.8 years (SD = 3.0). Gender distribution in the *PATH* group was 5 males (41.7%) and 7 females (58.3%); in the *ReCollect* group, 1 male (16.7%) and 5 females (83.3%); and in the OD group, 3 males (50.0%) and 3 females (50.0%). Across all groups, the total sample consisted of 24 participants, with a mean age of 41.1 years (SD = 12.9) and a mean education of 15.8 years (SD = 2.4), including 9 males (37.5%) and 15 females (62.5%). Statistical tests consisted of one-way ANOVAs for age and education, and chi-square tests were used for gender comparisons. There were no statistically significant differences between groups on age, education, or gender at baseline.

**TABLE 1:** Participant demographic characteristics by group.

Group	*n*	Age, mean (SD)	Education, mean (SD)	Female, n	Male, n
OD	6	38.7 (16.3)	14.8 (3.0)	3	3
*PATH*	12	43.2 (13.6)	16.2 (2.3)	7	5
*ReCollect*	6	39.5 (8.9)	15.7 (2.3)	5	1
Total*	24	41.1 (12.9)	15.8 (2.4)	15	9

Age and education are presented as means and standard deviations. Gender is presented as count (percentage) within each group. OD, Orientation Discrimination; PATH, Perceptual Attention THerapy; ReCollect intervention.

*Totals/means are calculated across all groups.

#### 3.2.2 Primary outcome measures

An assumption of the experiment was that participants would be successfully matched on the primary outcome measure of VWM. That assumption was tested with a one-way ANOVA, yielding a non-significant result, *F*(2, 9.98) = 2.5, *p* = 0.132 (see Table 2). While the variable of Processing Speed was not successfully matched, the remaining measures were not different at Time 1 (Pre-Tests).

**TABLE 2:** Cognitive pre-test comparisons across groups using one-way ANOVAs.

Measure	F	df1	df2	*P*
Visual working memory (Welch’s)	2.5	2	9.98	0.132
Processing speed (Welch’s)	33.5	2	9.72	<0.001***
Reading speed (Fisher’s)	2.92	2	21	0.076
Auditory working memory (Fisher’s)	1.12	2	21	0.344
Selective attention (Fisher’s)	1.27	2	21	0.302
Cognitive flexibility (Welch’s)	2.12	2	12.1	0.163

##### 3.2.2.1 Visual working memory

Descriptive statistics for VWM are reported in Table 3. To examine the impact of group assignment on changes in VWM performance, a 2 (Time: Pre, Post) × 3 (Group: *PATH*, *ReCollect*, OD) mixed-model ANOVA was conducted. Results from the mixed-model ANOVA indicated significant main effects of Group, *F*(2, 21) = 4.38, *p* = 0.026, η^2^*p* = 0.29, and Time, *F*(1, 21) = 14.67, *p* < 0.001, η^2^*p* = 0.41, as well as a significant Group × Time interaction, *F*(2, 21) = 5.67, *p* = 0.011, η^2^*p* = 0.35.

**TABLE 3:** Descriptive statistics for cognitive skills.

Intervention	*PATH*				*ReCollect*					OD		
Cognitive skills	Pre (SE)	Post (SE)	Post—Pre	%Change	Pre (SE)	Post (SE)	Post—Pre	%Change	Pre (SE)	Post (SE)	Post—Pre	%Change
Visual working memory (%)	31.1 (6.6)	80.0 (7.8)	48.9	157.2	61.8 (12.4)	74.9 (10.0)	13.1	21.2	29.0 (10.2)	36.7 (9.3)	7.7	36
Auditory working memory (%)	45.2 (7.6)	74.0 (6.5)	28.8	63.8	44.8 (10.8)	62.0 (14.0)	17.2	38.3	27.8 (6.9)	35.7 (14.0)	7.9	28.4
Processing speed (%)	37.4 (8.2)	65.8 (7.0)	28.4	75.7	59.3 (7.1)	70.2 (9.0)	10.9	18.3	4.3 (1.3)	9.9 (3.6)	5.6	127.2
Reading speed (words/min)	480 (71)	600 (87)	120	25	495 (97)	523 (98)	28	6	238 (36)	218 (58)	−20	−8
Selective attention (%)	46.5 (7.7)	66.9 (7.8)	20.4	43.8	47.7 (6.9)	58.0 (10.5)	10.3	21.7	29.0 (9.6)	28.5 (8.7)	−0.5	−1.7
Cognitive flexibility (%)	31.9 (8.8)	57.4 (7.6)	25.5	79.9	45.8 (5.4)	54.5 (6.3)	8.7	19.0	24.8 (9.6)	9.8 (3.1)	−15	−60.5

Planned pairwise comparisons of the change scores indicated that the *PATH* group demonstrated significantly greater improvements in VWM compared to both the *ReCollect* group (mean difference = 35.83, SE = 14.09, *p* = 0.019, Cohen’s *d* = 1.04) and the OD group (mean difference = 41.25, SE = 14.09, *p* = 0.008, Cohen’s *d* = 2.02). The *PATH* group improved considerably more (49% gain) than both the *ReCollect* (13% gain) and OD (8% gain) groups, with large effect sizes for both comparisons. These results are visually summarized in Figure 6a.

In summary, the hypothesis that group assignment would predict post-training gains in visual working memory was supported, with the *PATH* group exhibiting significantly greater improvements than both comparison groups.

#### 3.2.3 Secondary outcome measures

##### 3.2.3.1 Processing speed

Descriptive statistics revealed substantial baseline differences in the WAIS Processing Speed Index (PSI) scores across groups at Time 1 (Pre-Tests) (see Table 3). Specifically, the *PATH* group had a moderate PSI score at baseline (*M* = 37.42), the *ReCollect* group was higher (*M* = 59.33), and the OD group was markedly lower (*M* = 4.35), indicating clear non-equivalence prior to intervention. Because of this violation of initial group equivalence, a one-way ANCOVA was conducted on post-test PSI scores, using pre-test PSI as a covariate to statistically control for baseline differences.

The ANCOVA revealed a significant effect of group on the post-test Processing Speed score after controlling for pre-test scores, *F*(2, 21) = 15.41, *p* < 0.001, partial η^2^ = 0.59, indicating a large effect of treatment condition on adjusted post-test outcomes. Planned pairwise comparisons of the covariate-adjusted estimated marginal means showed that the *PATH* group demonstrated significantly greater post-intervention processing speed than the OD group, *t*(21) = 2.34, *p* = 0.029, with a large effect size (Cohen’s *d* = 1.31). The comparison between *PATH* and *ReCollect* approached significance, *t*(21) = 1.80, *p* = 0.087, with a moderately large effect size (Cohen’s *d* = 0.84). Taken together, these results indicate that, after controlling for initial processing speed differences, the *PATH* intervention produced the greatest gains (28.4% gain) and showed a significant advantage over the OD group (5.6% gain) with a large effect size.

##### 3.2.3.2 Reading speed

Descriptive statistics for Reading Speed are reported in Table 3. To examine whether group assignment influenced changes in computer-based reading speed (words per minute; WPM), a 2 (Time: Pre, Post) × 3 (Group: *PATH*, *ReCollect*, OD) mixed-model ANOVA was conducted. The analysis revealed a significant main effect of Group, *F*(2, 21) = 3.89, *p* = 0.037, partial η^2^ = 0.27, indicating overall differences in reading speed across groups. The main effect of Time did not reach significance, *F*(1, 21) = 2.97, *p* = 0.099, partial η^2^ = 0.12, suggesting that reading speed did not uniformly improve from pre- to post-test across all participants. The Group × Time interaction approached significance, *F*(2, 21) = 3.28, *p* = 0.058, partial η^2^ = 0.24, indicating that the magnitude of change over time differed by group.

To further explore this pattern, planned pairwise comparisons were conducted on the change scores (Post − Pre). The *PATH* group demonstrated a significantly greater increase in reading speed than the OD group, *t*(21) = 2.42, *p* = 0.025, with a large effect size (Cohen’s *d* = 1.17). Although the comparison between the *PATH* and *ReCollect* groups did not reach statistical significance, *t*(21) = 1.59, *p* = 0.126, the effect size was moderately large (Cohen’s *d* = 0.85), suggesting a meaningful advantage for *PATH*. As shown in Figure 6c, the *PATH* group demonstrated the largest gains in reading speed (120 words/min), significantly outperforming the OD group, which declined by 20 words/min (large effect size).

##### 3.2.3.3 Auditory working memory

Descriptive statistics for AWM are reported in Table 3. In the analysis of AWM, only an effect of Time emerged in the ANOVA, *F*(1, 21) = 15.08, *p* < 0.001, η^2^*p* = 0.42, indicating that auditory working memory scores increased from pre- to post-test across groups. There was no significant main effect of Group, *F*(2, 21) = 3.15, *p* = 0.064, η^2^*p* = 0.23, and no significant Group × Time interaction, *F*(2, 21) = 2.02, *p* = 0.158, η^2^*p* = 0.16, suggesting that the magnitude of improvement over time did not significantly differ between groups. The hypothesis that group assignment would predict post-training gains in auditory working memory was partially supported, with the PATH group exhibiting the most pronounced improvement following intervention training (see Figure 6d). The *PATH* group improved considerably more in AWM (28.8% gain) than the OD group (7.9% gain). This comparison approached significance, *t*(21) = 1.96, *p* = 0.064, with a large effect size (*d* = 1.09), suggesting that *PATH* may produce substantially greater gains than OD, though the results must be interpreted with caution. The *PATH* vs. *ReCollect* contrast was nonsignificant, *t*(21) = 1.09, *p* = 0.290, with a medium effect size (*d* = 0.50).

##### 3.2.3.4 Selective attention

Descriptive statistics for Selective Attention are reported in Table 3. A 2 (Time: Pre, Post) × 3 (Group: *PATH*, *ReCollect*, OD) mixed-model ANOVA revealed a significant main effect of Group, *F*(2, 21) = 3.65, *p* = 0.0437, η^2^*p* = 0.26, indicating overall differences in Selective Attention between intervention groups. The main effect of Time was not significant, *F*(1, 21) = 3.37, *p* = 0.0807, η^2^*p* = 0.14, suggesting that gains in Selective Attention from pre- to post-test were not consistent across all participants. The Group × Time interaction was also nonsignificant, *F*(2, 21) = 1.37, *p* = 0.275, η^2^*p* = 0.12, indicating that the amount of change over time did not differ significantly between groups.

In order to facilitate comparison with other dependent measures, planned comparisons were carried out. The *PATH* vs. OD contrast approached significance, *t*(21) = 1.64, *p* = 0.117, with a large effect size (*d* = 1.12), indicating that *PATH* produced notably greater improvement than OD, although this difference did not reach conventional statistical significance. The *PATH* vs. *ReCollect* comparison was nonsignificant, *t*(21) = 0.79, *p* = 0.440, with a small-to-medium effect size (*d* = 0.32). As shown in Figure 6e, the *PATH* group improved considerably more in Selective Attention (20.4% gain) than the OD group (0.5% decline). Though not statistically significant, this difference appears practically meaningful.

##### 3.2.3.5 Cognitive flexibility

Descriptive statistics for Cognitive Flexibility are reported in Table 3. To examine whether group assignment influenced changes in Cognitive Flexibility, a 2 (Time: Pre, Post) × 3 (Group: *PATH*, *ReCollect*, OD) mixed-model ANOVA was conducted. Results revealed a significant main effect of Group, *F*(2, 21) = 4.45, *p* = 0.025, η^2^*p* = 0.30, indicating overall group differences in performance. The Group × Time interaction was also significant, *F*(2, 21) = 8.28, *p* = 0.002, η^2^*p* = 0.44, demonstrating that the pattern of change from pre- to post-test differed across the three groups. However, the main effect of Time was not significant, *F*(1, 21) = 2.20, *p* = 0.153, η^2^*p* = 0.09, suggesting that improvement did not occur uniformly across all participants regardless of group.

Planned pairwise comparisons of change scores showed that the PATH group improved significantly more than the OD group (mean difference = 40.46, *SE* = 9.98, *p* < 0.001, *d* = 2.03), reflecting a very large effect size. In contrast, the difference between the *PATH* and *ReCollect* groups did not reach statistical significance (mean difference = 16.79, *SE* = 9.98, *p* = 0.107, *d* = 0.85), although the effect size was in the large range. These results are visually summarized in Figure 6f.

In summary, the hypothesis that group assignment would influence gains in cognitive flexibility was supported. The *PATH* group demonstrated the largest improvement following intervention training (25.5% gain), significantly outperforming the OD group (15% decline) with a very large effect size. The *PATH* group also showed a meaningful advantage over the *ReCollect* group (8.7% gain) with a large effect size, though this difference was not statistically significant.

##### 3.2.3.6 Adult dyslexia test reading proficiency level

The analyses did not indicate any significant differences in Group or Time for the ADT-RPL.

#### 3.2.4 Quality of life measures

##### 3.2.4.1 Rivermead post-concussion questionnaire

**FIGURE 7:**
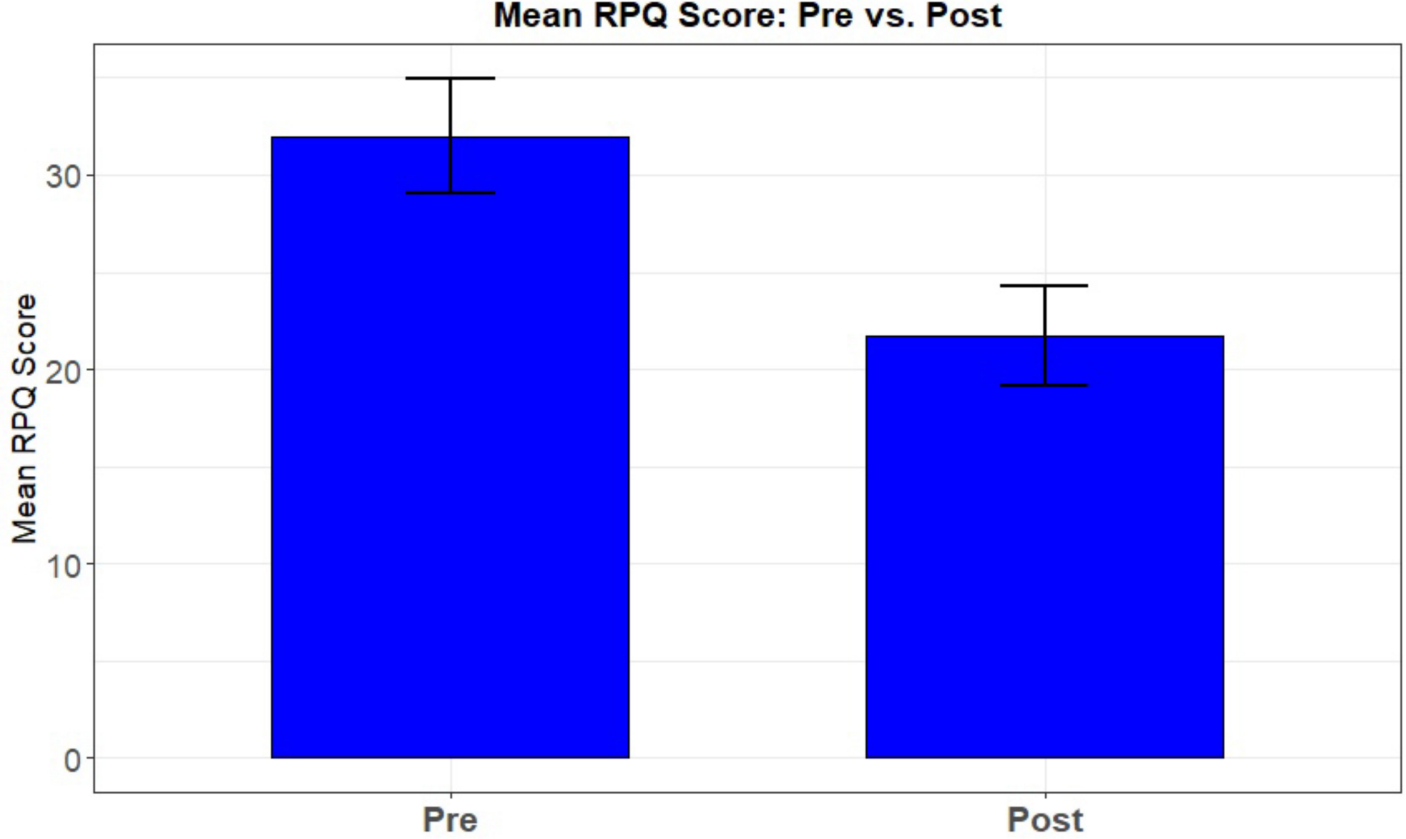
Mean difference in RPQ-13 (Post—Pre) collapsed across groups.

The RPQ-3 assesses three core post-concussive symptoms (headaches, dizziness, and nausea). A 2 (Time: Pre, Post) × 3 (Group: *PATH*, *ReCollect*, OD) mixed-model ANOVA revealed a significant main effect of Time, *F*(1, 21) = 6.81, *p* = 0.016, η^2^*p* = 0.24, indicating that RPQ-3 symptom severity decreased from pre- to post-test across participants. There was also a significant main effect of Group, *F*(2, 21) = 3.59, *p* = 0.046, η^2^*p* = 0.25, reflecting overall differences in symptom severity among the three interventions. However, the Group × Time interaction was not significant, *F*(2, 21) = 1.17, *p* = 0.329, η^2^*p* = 0.10, indicating that the amount of improvement over time did not differ significantly by group. Planned comparisons showed that *PATH* did not differ significantly from *ReCollect* (*p* = 0.587, *d* = 0.28) or OD (*p* = 0.141, *d* = 0.77) in symptom change. Thus, although all groups showed reductions in RPQ-3 symptoms over time, no intervention produced significantly greater improvement than the others. The pre-post difference scores collapsed across groups are shown in Figure 7.

##### 3.2.4.2 SF-36 health survey

To assess changes in perceived role limitations due to emotional problems, the SF-36 Role Emotional domain was examined across the *PATH*, *ReCollect*, and OD groups. This domain evaluates the extent to which emotional problems interfere with daily activities such as work or other routine responsibilities; for example, items ask whether participants cut down time on activities, accomplished less than desired, or performed less carefully because of emotional difficulties.

**FIGURE 8:**
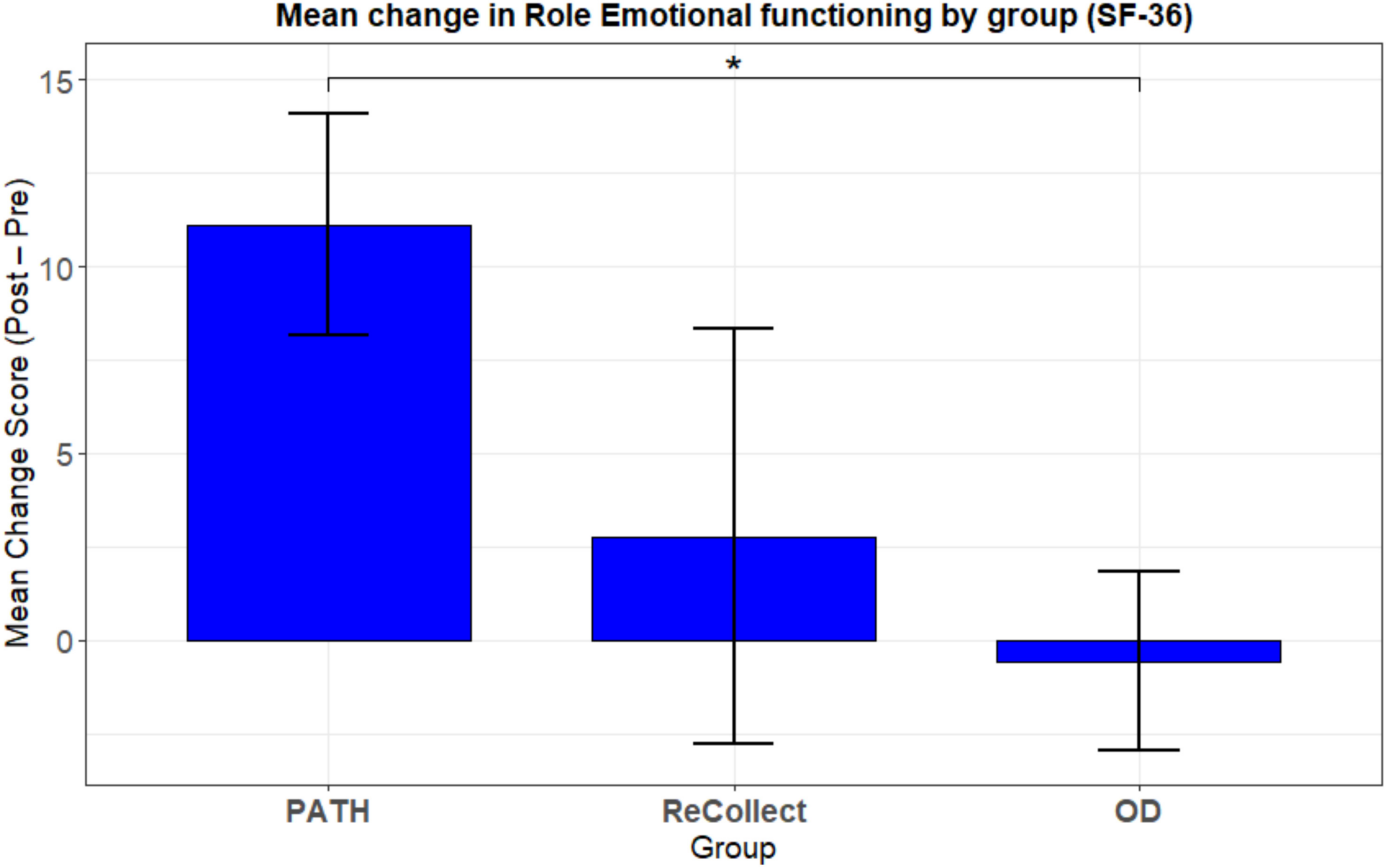
Mean change in Role Emotional functioning by group (SF-36).

A 2 (Time: Pre, Post) × 3 (Group: *PATH*, *ReCollect*, OD) mixed-model ANOVA revealed a significant main effect of Group, *F*(2, 21) = 3.77, *p* = 0.040, η^2^*p* = 0.26, indicating overall differences in Role Emotional functioning across the three conditions, (see Figure 8). The main effect of Time approached significance *F*(1, 21) = 3.96, *p* = 0.060, η^2^*p* = 0.16, suggesting a trend toward improvement from pre- to post-test across groups. However, the Group × Time interaction did not reach traditional significance, *F*(2, 21) = 2.96, *p* = 0.074, η^2^*p* = 0.22, but showed a moderate effect size, warranting follow-up comparisons to examine specific group differences in change over time.

Planned contrasts on change scores (Post—Pre), using OD as the reference group, showed that the *PATH* group improved significantly more than the OD group [estimate = 11.69, SE = 5.18, *t*(21) = 2.26, *p* = 0.035, *d* = 1.40]. In contrast, the difference between *ReCollect* and OD was not significant [estimate = 8.33, SE = 5.18, *t*(21) = 1.61, *p* = 0.123, *d* = 0.69].

In summary, while overall group differences and time-related improvements were modest, only the *PATH* group demonstrated a statistically and clinically meaningful reduction in role limitations due to emotional problems relative to the OD group. The *ReCollect* group showed a small, non-significant improvement. These findings suggest that *PATH* produced the most consistent gains in emotional role functioning following intervention training.

#### 3.2.5 Analysis of effect sizes

Given the relatively small sample size (*n* = 24), it is important to determine whether our study was adequately powered to detect group effects on our outcome measures. Across all primary outcomes, partial η^2^ values ranged from small to large, with Group effects spanning η^2^*p* = 0.23–0.59 (small-to-medium to very large) and Group × Time interactions ranging from η^2^*p* = 0.12 to 0.44 (small-to-large). This pattern indicates notable variability in the magnitude of group differences and differential change over time across measures.

Similarly, Cohen’s d values for planned contrasts showed consistently medium to very large effects. Specifically, *PATH* vs. *ReCollect* ranged from *d* = 0.32 to 1.04 (small-to-medium to large), and *PATH* vs. OD ranged from *d* = 1.09 to 2.03 (large to very large) across measures (see Table 4). Notably, even when statistical significance was not achieved (e.g., in Selective Attention and Auditory Working Memory), effect sizes remained large for *PATH* vs. OD, indicating potentially meaningful advantages for *PATH* despite limited power.

**TABLE 4:** Effect size summary for outcome measures.

Measure	Time η*p*	Group η^2^*p*	Interaction η^2^ *p*	PATH vs. ReCollect (d)	PATH vs. OD (d)
Visual working memory	0.41	0.29	0.35	1.04	2.02
Processing speed (ANCOVA)	–	0.59	–	0.84	1.31
Reading speed	0.12	0.27	0.24	0.85	1.17
Auditory working memory	0.42	0.23	0.16	0.50	1.09
Selective attention	0.14	0.26	0.12	0.32	1.12
Cognitive flexibility	0.09	0.30	0.44	0.85	2.03
SF-36 emotional role	0.16	0.26	0.22	0.69	1.40
RPQ (symptoms)	0.24	0.25	0.10	0.28	0.77

Because planned contrasts were tested between *PATH* (*n* = 12) and each comparator (*n* = 6), we evaluated statistical power for those pairwise tests using the observed effect sizes. For *PATH* vs. OD, contrasts with very large effects (e.g., Visual Working Memory *d* = 2.02; Cognitive Flexibility *d* = 2.03; Emotional Role *d* = 1.40) were well powered (≈0.75–0.97). In Auditory Working Memory (*d* = 1.09) and Selective Attention (*d* = 1.12), effects were also large, indicating practical importance, but power was likely moderate at best, contributing to nonsignificant p-values.

In contrast, *PATH* vs. *ReCollect* effects in the small-to-large range (e.g., *d* ≈ 0.32–1.04 across outcomes) yielded limited power (≈0.10–0.50), helping explain why several meaningful effect sizes did not reach statistical significance. A prospective calculation for this contrast structure (*PATH n* = 12 vs. comparator *n* = 6; α = 0.05, two-tailed) was used to determine the minimal detectable effect size (MDE)—the smallest true effect size likely to be detected with 80% power. This analysis indicated an MDE of approximately *d* = 1.48, implying that effects smaller than ∼1.5 would often be underpowered with the current group sizes.

Taken together, contrasts against OD were generally well powered when effects were very large, whereas contrasts against *ReCollect* were underpowered unless effects approached or exceeded d ≈ 1.5. Importantly, the consistent pattern of large effect sizes favoring *PATH*—especially in Visual Working Memory, Processing Speed, Reading Speed, Auditory Working Memory, Selective Attention, Cognitive Flexibility, and Emotional Role (Table 4)—supports the practical significance of the *PATH* intervention even when statistical significance was not always achieved.

## 4 Discussion

This study investigated the efficacy of *PATH* training in ameliorating cognitive deficits following mild traumatic brain injury (mTBI). Participants were randomized to three interventions: *PATH*, *ReCollect*, or Orientation Discrimination (OD). Cognitive performance was evaluated across six domains: Visual Working Memory (VWM), Auditory Working Memory (AWM), Processing Speed, Reading Speed, Selective Attention, and Cognitive Flexibility. Consistent with the Results and the Analysis of Effect Sizes, *PATH* training produced the most substantial improvements across domains, with effects that were not only statistically reliable in several measures but also practically large. Across outcomes, *PATH* showed consistently large to very large effect sizes relative to OD and medium-to-large effects relative to *ReCollect*, underscoring meaningful clinical gains even when some *p*-values were marginal due to limited power.

*PATH* training produced the most substantial improvements across all cognitive domains. VWM showed remarkable enhancement (49% improvement) in the *PATH* group, dramatically exceeding gains in *ReCollect* (13%) and OD (8%) groups. These behavioral gains were accompanied by large contrasts in effect size (e.g., large vs. both comparators), reinforcing the robustness of the VWM advantage in *PATH*. AWM and Selective Attention improvements, while more modest, were substantially greater in the *PATH* group compared to OD, indicating broader transfer to auditory processing domains. Importantly, even when not consistently meeting conventional significance thresholds, the *PATH* vs. OD contrasts for AWM and Selective Attention were large (d ≈ 1.1), supporting practical significance and suggesting that limited statistical power, rather than negligible effects, likely constrained some inferential tests. The *PATH* group also demonstrated significantly superior performance in Processing Speed (particularly Digit Symbol Coding), Reading Speed, and Cognitive Flexibility relative to the OD group. Notably, Selective Attention improvements, measured by the Stroop test (DKEFS Inhibition), were characterized by faster response times and reduced errors. Together with the effect size profile across domains, these findings suggest that *PATH* training yields broad and clinically meaningful improvements that extend beyond vision-specific tasks. These findings collectively suggest that *PATH* training recalibrates fundamental sensory timing deficits while enhancing higher-order cognitive functions.

### 4.1 Theoretical framework and mechanistic support

These results align with research demonstrating that magnocellular pathway damage within the dorsal visual stream contributes to chronic visual timing and attentional impairments following mTBI (Shenton et al., 2012; Dean et al., 2012). Disruptions in dorsal stream functionality result in persistent VWM and processing speed deficits due to impaired sensory integration (Hebert and Filley, 2022). The current findings support this framework: *PATH*’s direct targeting of low-level motion discrimination facilitated significant VWM and Processing Speed improvements, suggesting that dorsal stream timing recalibration provides the neural foundation necessary for higher-order cognitive processes. Moreover, the consistently large effect sizes observed for *PATH*—particularly relative to OD and often medium-to-large relative to *ReCollect*—indicate meaningful treatment impact that is congruent with dorsal stream timing restoration. The presence of large effects in AWM and Selective Attention, even when *p*-values were marginal, is consistent with a mechanistic account in which genuine neural changes are partially masked by limited statistical power rather than by weak intervention effects.

The robust processing speed gains in *PATH* participants corroborate emerging evidence that rehabilitative programs optimizing dorsal stream function enhance visual discrimination speed and dynamic stimulus integration (Krucoff et al., 2016). Conversely, the OD intervention, which targets ventral stream processing, yielded minimal improvements in VWM, AWM, Processing Speed, Reading Speed, Selective Attention, and Cognitive Flexibility, despite its established benefits for object perception and form recognition (Goodale and Milner, 1992). This contrast underscores the limited generalizability of ventral stream training to cognitive domains requiring precise spatiotemporal processing.

AWM improvements in the *PATH* group are consistent with neuroimaging findings suggesting that interventions addressing foundational sensory timing deficits enhance connectivity between posterior sensory and anterior executive control regions (Iacoboni, 2005). However, the smaller effect size for AWM compared to VWM and Processing Speed indicates that *PATH*’s primary impact is most pronounced for visually mediated tasks, likely reflecting the greater reliance of VWM and Processing Speed on dorsal stream reorganization.

Selective Attention enhancements in the *PATH* group align with research demonstrating that recalibrating sensory timing reduces cognitive effort required for motion-related cue processing, thereby liberating attentional resources for higher-order tasks (Petersen and Posner, 2012). The minimal improvements in the OD group suggest that ventral stream pattern discrimination alone is insufficient for modulating attention and inhibitory control—cognitive processes requiring precise spatiotemporal synchronization.

Traumatic brain injury induces significant cortical modifications that contribute to cognitive impairment independent of initial injury severity. These alterations include reduced cortical thickness, disrupted functional connectivity, and persistent neuroinflammatory responses. TBI patients exhibit decreased cortical thickness in regions associated with executive function and working memory, particularly in the prefrontal cortex (Caeyenberghs et al., 2017), shown by MRI and fMRI imaging. Resting-state fMRI studies reveal widespread functional connectivity disruptions within the default mode network (DMN), correlating with observed cognitive deficits (Hillary et al., 2015). Chronic neuroinflammation, characterized by persistent microglial activation and elevated pro-inflammatory cytokines, contributes to long-term cognitive decline in TBI survivors (Simon et al., 2017). These findings indicate that post-TBI cognitive impairment results not solely from mechanical injury forces but from progressive cortical modifications that may exacerbate deficits over time. Taken together, the large behavioral effects favoring *PATH* across multiple domains and the theoretical account of dorsal stream timing recalibration converge to support the interpretation that *PATH*’s benefits are both mechanistically grounded and practically meaningful, even where significance tests were constrained by sample size. The effect size profile—large advantages for *PATH* vs. OD across domains and medium-to-large advantages vs. *ReCollect*—dovetails with the neural mechanism of enhanced dorsal stream timing, providing convergent evidence that *PATH* alters both function and neurophysiology in ways that generalize to cognition and everyday performance.

#### 4.1.1 MEG evidence of functional reorganization

A unique contribution of this study is the inclusion of neurophysiological data using MEG during both task performance (N-back) and resting state. Consistent with previous findings (Lawton and Huang, 2019; Lawton, 2025), we observed significantly stronger post-treatment responses in bilateral dorsolateral prefrontal cortex (dlPFC), posterior parietal cortex (PPC), anterior cingulate cortex (ACC), superior occipital gyri, and—newly identified—left anterior temporal lobe, left hippocampus, primary motor cortex, and cerebellum in *PATH*-treated individuals compared to *ReCollect* (*p* = 0.001) (Figure 4). These enhancements encompass vision, attention, executive control, and memory-related neurocircuitry.

Critically, our MEG results demonstrate both increased task-evoked synchronization in the canonical working memory network (dlPFC, ACC, parietal cortex) and marked reduction in gamma-band “noise” during resting-state. Greater gamma-band noise throughout the brain was associated with poorer cognition, specifically executive functioning and visuospatial processing (Huang et al., 2020). Less gamma-band noise, which improves both the accuracy of gamma feedback by improving the signal to noise ratio (Hoogenboom et al., 2005) and the inhibitory processes necessary for left-right movement discrimination (as demonstrated by Levinson and Sekuler, 1975), originated from the vision networks (bilateral superior occipital gyri) (Figure 5). This dual pattern suggests that *PATH*+DM neurotraining enhances task-specific neural recruitment while normalizing background oscillatory activity—a finding consistent with animal and computational models of GABAergic network dysfunction (Carlén et al., 2012). This functional reorganization supports the interpretation that *PATH* facilitates neural recovery through enhanced evoked synchronization and restoration of inhibitory tone.

#### 4.1.2 Mechanistic convergence with preclinical models

The observed neurophysiological effects align remarkably with the mechanistic framework established by Carlén et al. (2012), who demonstrated in animal models that NMDAR signaling in fast-spiking parvalbumin (PV) interneurons is essential for generating and synchronizing gamma oscillations—rhythms underlying working memory, attention, and cognitive flexibility. Following mTBI, injury to GABAergic interneurons impairs inhibition, leading to elevated resting-state gamma power (“noise”) and diminished task-evoked synchrony. Carlén et al. (2012) demonstrated that restoring NMDA function in PV interneurons enables recovery of both baseline and evoked gamma activity, critically restoring cognitive performance.

Our MEG findings directly mirror this pattern: mTBI patients post-*PATH* showed reduced spontaneous gamma activity at rest coupled with enhanced, well-synchronized task-evoked responses during working memory demands. These results provide rare *in vivo* human evidence that interventions targeting sensory timing and inhibitory synchrony—such as *PATH*—may effect neurophysiological changes predicted by preclinical models, potentially through normalizing GABAergic network function and improving NMDAR signaling.

This convergence between animal models, computational theory, and human MEG data strengthens the case that gamma oscillatory dynamics, regulated by PV interneuron networks, serve as both biomarkers and mediators of cognitive rehabilitation outcomes in mTBI. The identification of novel neuroplastic changes in anterior temporal lobe, hippocampus, motor cortex, and cerebellum may represent compensatory pathways engaged by *PATH*+DM neurotraining, extending beyond primary visual-attentional networks to encompass broader cognitive and motor systems.

### 4.2 Individual variability in treatment response

Individual differences in treatment response likely stem from multiple factors including baseline impairment severity, protocol adherence, and neurophysiological variability. Research indicates that pre-existing cognitive deficits significantly influence intervention responsiveness, with more severely impaired individuals often demonstrating greater gains due to larger improvement margins (Mahncke et al., 2021). Additionally, participant engagement and adherence correlate with more robust cognitive improvements (Barnes et al., 2009).

Neurophysiological factors, including individual differences in cortical plasticity and compensatory neural mechanisms, also contribute to recovery variability. Functional neuroimaging studies indicate that patients with greater baseline functional connectivity in dorsal stream networks exhibit enhanced learning capacity and cognitive recovery following TBI (Wu et al., 2015). Variations in genetic and neurochemical profiles, such as brain-derived neurotrophic factor (BDNF) expression differences, have been linked to differential neuroplastic responses and rehabilitation outcomes (Treble-Barna et al., 2024).

Psychosocial factors, including motivation, stress levels, and mental health status, may further mediate intervention efficacy. Individuals with higher intrinsic motivation and lower anxiety levels tend to engage more effectively with cognitive training, leading to greater executive function and working memory improvements (Ansari, 2015). Future research should identify specific predictors of treatment response to enable more tailored intervention strategies for optimizing mTBI cognitive rehabilitation.

### 4.3 Quality of life and functional outcomes

Preliminary findings suggest promising quality-of-life enhancements, as measured by the Rivermead Post-Concussion Symptoms Questionnaire (RPQ) and SF-36 Role Limitations due to Emotional Problems (RE) subscale. All groups demonstrated significant RE score improvements from pre- to post-test, indicating meaningful reductions in perceived emotional role limitations. This pattern was mirrored in RPQ outcomes, showing substantial symptom reduction following training.

While no significant group differences were detected in some functional outcomes, *PATH* participants demonstrated the largest RE score gains, suggesting unique potential for alleviating emotional role limitations. These preliminary results warrant further investigation and *PATH* methodology refinement to optimize quality-of-life impacts. Larger sample sizes and more controlled experimental designs will be essential to fully elucidate distinct *PATH* training benefits. Importantly, the effect size profile complements these observations: large *PATH* vs. OD effects in Emotional Role functioning and robust effects across multiple cognitive domains support the practical significance of *PATH*’s impact on day-to-day functioning, even where significance tests were occasionally constrained by sample size.

### 4.4 Limitations

Several limitations temper interpretation of these findings. Although the present sample size exceeded that of our previous mTBI rehabilitation studies (Lawton and Huang, 2019; Lawton, 2025), it remains modest in absolute terms. Importantly, this sample size is consistent with the empirical literature on software-based cognitive rehabilitation, in which final analyzed samples typically range from 4 to 30 participants, with a mean of approximately 16.1 completers across published trials (Barnes et al., 2009; Bergquist et al., 2008; Chandler et al., 2017; Kesler et al., 2011; Verhelst et al., 2017; Westerberg et al., 2007; Zickefoose et al., 2013). Indeed, small samples are the norm rather than the exception in this field, particularly when interventions involve multiple training sessions combined with neuroimaging or other resource-intensive assessments. The high cost of MRI/EEG acquisition, limited scanner access, participant burden across repeated sessions, and the need for highly trained personnel frequently constrain recruitment and retention, resulting in underpowered studies across the literature.

While our sample compares favorably to these prior efforts, the limited statistical power still restricts generalizability. Several outcomes (e.g., Reading Speed, Cognitive Flexibility, Selective Attention, and Auditory Working Memory) showed trends toward improvement but did not consistently reach conventional significance thresholds when comparing *PATH* to *ReCollect* groups. Crucially, many of these contrasts nonetheless exhibited medium-to-large or large effect sizes (e.g., *PATH* vs OD in AWM and Selective Attention), suggesting that some nonsignificant findings likely reflect underpowering rather than negligible treatment effects. These patterns are encouraging but require validation in larger, adequately powered samples. To address these limitations and more fully elucidate the mechanisms and clinical impact of *PATH* neurotraining, a Phase II clinical trial is planned, which will allow for increased sample size, stronger statistical inference, and broader generalizability.

### 4.5 Future directions

Future research priorities include:

- *Multi-site randomized controlled trials* with adequate power to replicate and extend these findings.
- *Longitudinal follow-up studies* to assess cognitive and neurophysiological change durability.
- *Multimodal imaging and genetic marker integration* to identify response predictors and underlying mechanisms.
- *Development of ecologically valid outcome measures* that capture functional recovery and quality-of-life improvement.
- *Hybrid intervention protocols* combining *PATH* with other evidence-based rehabilitation strategies to maximize benefit across diverse mTBI profiles.

## 5 Conclusion

This study provides compelling evidence that *PATH* neurotraining, when delivered as a targeted mTBI intervention, yields substantial cognitive functioning improvements—most notably in visual working memory, processing speed, reading speed, and cognitive flexibility, but also in auditory working memory and selective attention. These behavioral gains are supported by converging neurophysiological evidence from MEG, demonstrating enhanced task-evoked activation and normalization of resting-state gamma oscillatory activity within distributed vision, attention, and executive control networks encompassing occipital, temporal, cingulate, parietal, prefrontal, motor, and cerebellar regions.

These results replicate and extend prior findings (Lawton and Huang, 2019; Lawton, 2025) while substantiating a mechanistic model in which GABAergic network synchronization restoration, particularly via parvalbumin interneuron function, underpins both cognitive recovery and neural plasticity following mTBI. The convergence between animal models, computational predictions, and in vivo human neuroimaging data strengthens gamma oscillations’ position as biomarkers—and potential mediators—of successful neurorehabilitation.

Quality-of-life improvements, including post-concussive symptom reductions and enhanced emotional role functioning, further highlight the *PATH* protocol’s broader clinical relevance. Across cognitive domains, *PATH* consistently produced large or very large effect sizes relative to OD and medium-to-large effects relative to *ReCollect*, even when statistical significance was not always reached. These findings emphasize the practical and clinical importance of *PATH*’s benefits in the context of small-sample neurorehabilitation research and align closely with mechanistic evidence for dorsal stream timing recalibration and improved inhibitory synchrony. While several limitations warrant consideration—including modest sample size, limited scope of active control conditions, and the use of standardized but sometimes non-ecological outcome measures—the consistent pattern of large behavioral effect sizes and converging neurophysiological evidence suggests that *PATH*’s benefits are robust rather than incidental. Future Phase II trials with larger, more diverse samples and more sensitive, ecologically valid outcome measures will be essential to determine the generalizability, durability, and mechanisms of the effects observed here.

Collectively, these findings position *PATH* neurotraining as a promising, evidence-based and mechanistically-informed intervention for ameliorating mTBI’s cognitive and neurophysiological sequelae. *PATH* neurotraining represents a paradigm shift by targeting underlying mTBI etiology rather than addressing symptoms in isolation. As research matures, integrating behavioral performance, neuroimaging markers, and translational biomarkers will be vital for personalizing rehabilitation protocols, enhancing scalability, and optimizing long-term functional outcomes for individuals affected by traumatic brain injury.

## Data Availability

The datasets presented in this study can be found in online repositories. The names of the repository/repositories and accession number(s) can be found at: OSF (Open Sciences Framework) data repository for the behavioral data (https://osf.io/ uz7qm/overview?view_only=3231625cfdbd419daf2a8cb40a7e6533).

## Footnotes

1
https://www.oepf.org/product/dyslexia-determination-testing-and-diagnosis/

2
https://app.pathtoreading.com

3
http://afni.nimh.nih.gov
