# Correction: Retraining dorsal visual pathways improves cognitive skills and executive control networks following mild traumatic brain injury

**DOI:** 10.3389/fnhum.2025.1764677

**Published:** 2025-12-29

**Authors:** Frontiers Production Office

**Affiliations:** Frontiers Media SA, Lausanne, Switzerland

**Keywords:** TBI, cognitive rehabilitation, visual timing, improve cognitive skills, visual working memory, memory recruitment and storage, processing speed, attention

The reference for (Lawton, 2025) was erroneously written as “Lawton, T., Shelley-Tremblay, J., Lee, R. R., and Huang, M. X. (2025a). Retraining dorsal visual pathways improves cognitive skills after a mild traumatic brain injury. J. Clin. Med. 14:2273. doi: 10.3390/jcm14072273” and “Lawton, T., Shelley-Tremblay, J., Lee, R. R., and Huang, M. X. (2025b). Retraining dorsal visual pathways improves cognitive skills after a mild traumatic brain injury. J. Clin. Med. 14:2273. doi: 10.3390/jcm14072273”. It should be: “Lawton, T. (2025). Retraining Dorsal Visual Pathways Improves Cognitive Skills and Improves Executive Control Networks. Washington, DC: U.S. Patent and Trademark Office, Filed on November 15, 2025.”

The acknowledgments statement was omitted from article. It should read:

Acknowledgments: We wish to thank Annemarie Angeles Quinto, Hayden Hansen, Isabel Rodriguez, and Cameron Finch for collecting the data presented in this study. We thank Federico Rossano in the Cognitive Science Department at UCSD for serving as the faculty sponsor for Independent Research course credit for all of our UCSD students who collected the pre- and post-neuropsychological data. We thank Alan Shahtaji, Mohammed Ahmed, Shaul Saddick, Maysa Nagi, Sarah Kalina, Heike Kessler-Heiberg, Brandi Bass, Spine and Sport Physical Therapy, the San Diego Brain Injury Foundation (SDBIF), and the UCSD clinical trials website for mTBI patient referrals.

The original version of this article has been updated.

