# Supplementary material for: Retraining dorsal visual pathways improves cognitive skills and executive control networks following mild traumatic brain injury
Source: Front Hum Neurosci. 2025 Dec 9;19:1698605. doi: 10.3389/fnhum.2025.1698605 (PMC12722797; doi:10.3389/fnhum.2025.1698605)
Supplement: Supplementary file 1 [file Data_Sheet_1.pdf]

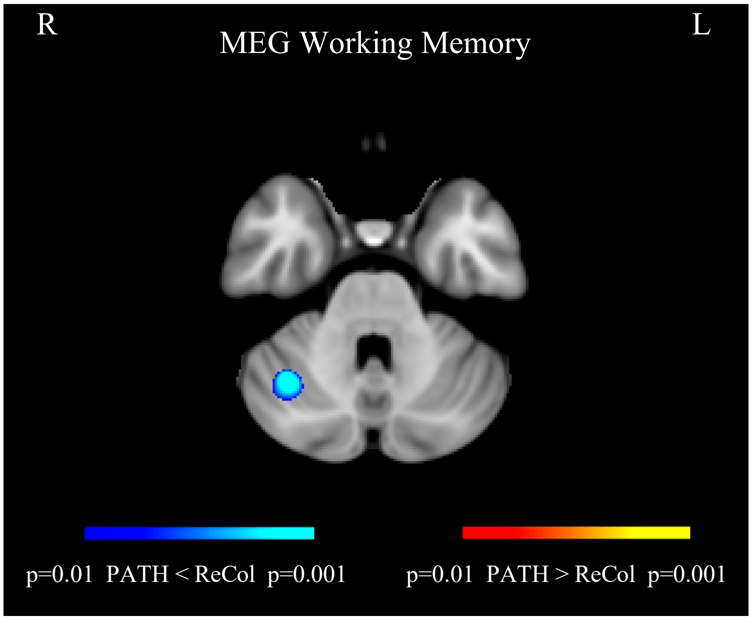


**Figure S1**. Baseline (pre-treatment) working memory N-back MEG exam: subjects in the PATH mTBI group showed lower response in the right cerebellum than those in the ReCollect mTBI group.
